# Optimization of Batch Centralized Health Examination Scheduling Considering Inter‐Item Transition Time and Sex Differences: An Empirical Optimization Study

**DOI:** 10.1002/hsr2.72912

**Published:** 2026-08-06

**Authors:** Mengdi Lv, Xiaoming Zhao, Fangxu Sun, Yang Kong, Xin Wang

**Affiliations:** ^1^ School of Health Management Shandong Medical and Pharmaceutical University (Former Binzhou Medical College) Yantai China; ^2^ Center of Physical Examination, Shandong Medical and Pharmaceutical University (Former Binzhou Medical College) Affiliated 970 Hospital of the PLA Joint Logistic Support Force Yantai China; ^3^ Department of Clinical Laboratory & Medical Service Training Shandong Medical and Pharmaceutical University (Former Binzhou Medical College) Affiliated 970 Hospital of the PLA Joint Logistic Support Force Yantai China

**Keywords:** batch centralized physical examination, GA, group physical examination, scheduling optimization

## Abstract

**Background and Aims:**

With increasing health awareness among Chinese residents, the demand for health examinations has continued to grow, placing mounting pressure on hospitals and health examination centers. This problem is particularly evident in group batch health examinations, where a large instantaneous flow of examinees and numerous examination items can easily lead to disorder in the examination process. This study aimed to optimize scheduling for this type of health examination, improve examination efficiency, and shorten total examination time.

**Methods:**

A health examination scheduling optimization model was developed with the objective of minimizing the maximum examination duration. The model incorporated path‐dependent transition time between examination items and sex‐related differences, and a genetic algorithm was designed to obtain scheduling solutions. For special group examination populations with precedence constraints among examination items, the model was modified, and a controlled experiment was designed to quantitatively evaluate the effects of introducing constraints on algorithm efficiency and optimization performance. Finally, experimental analyses were conducted, including model verification using small‐scale benchmark examples, case analysis, AnyLogic simulation, and sensitivity analysis, to validate the effectiveness of the model and algorithm and the stability of the solutions.

**Results:**

Introducing constraints had only a minor effect on algorithm efficiency, although it reduced the optimization effect. Model verification showed that the differences in examination duration and transfer time were both 0, and the scheduling results satisfied the inter‐item constraints, indicating that the model correctly implemented the predefined scheduling logic. In the case analysis, the optimized scheduling scheme yielded a maximum examination duration of 197.06 min, while the simulation result was 195.57 min, with a difference of only 1.49 min. Compared with the pre‐optimization duration of 220 min, the simulation result represented an 11.10% reduction (24.43 min/220 min), confirming the effectiveness of the model and algorithm. Sensitivity analysis showed that, when service durations varied within ±5% and ±10%, most item‐duration changes except ultrasound could be absorbed by the system buffer and had limited influence on the total examination duration, demonstrating a certain degree of stability in the model and algorithm solutions.

**Conclusion:**

The proposed mathematical programming model and genetic algorithm can significantly improve scheduling efficiency for batch centralized health examinations, substantially shorten overall examination duration, and have strong practical application value.

## 1 Introduction

With socioeconomic development and improvements in living standards, health awareness among Chinese residents has gradually increased. This trend was further accelerated during the COVID‐19 pandemic, leading more people to undergo regular health examinations. According to data from the National Health Commission, the number of health examinations in China reached 503 million in 2022, compared with 238 million in 2010, representing an increase of 52.68%. Against this background, hospitals and health examination institutions are facing increasing workload. Although appointment‐based examinations and other pre‐examination triage methods have been adopted, examinee routes during the examination process often remain disordered.

According to the 2022 annual report of Meinian Onehealth, a leading chain enterprise in the health examination industry, group clients accounted for 83% of its customers, highlighting the dominant role of group health examinations in market demand. Group health examinations include batch centralized examinations uniformly organized by organizations and decentralized examinations in which individuals visit the examination site within a specified time window. Compared with decentralized group examinations and individual examinations, batch centralized examinations are characterized by large instantaneous patient flows and numerous examination items. Disorderly examination arrangements result in resource mismatches, chaotic workflows, and severe congestion, increasing examinees' time costs while reducing the efficiency of medical resource utilization. At the current stage, given the manufacturing cost constraints of medical equipment, relying on additional equipment to solve this problem is impractical. Therefore, it is essential to reasonably plan the scheduling of such group health examinations, achieve high‐quality matching between existing medical resources and examinees, shorten examination duration, and improve the examinee experience.

In group health examination processes, examination items usually have no strict order constraints, or only partial precedence constraints. Examinees may flexibly adjust their examination sequence according to on‐site conditions and personal needs. In addition, the same type of examination item is often supported by multiple functionally equivalent devices, and different examinees may not need to complete all examination items because of differences in examination packages. Given these features, mixed scheduling models can be constructed to characterize different stages or constraint types in the examination process; however, this approach usually requires artificial segmentation of the process and the introduction of additional structural assumptions, thereby increasing model complexity. Conversely, the flexible job‐shop scheduling problem (FJSP) can describe cases with precedence constraints among examination items, but it requires all operations of each job to be predefined in a uniform sequence. This is not fully consistent with group health examinations, where the order of examination items is arbitrary and only local constraints may exist. In contrast, the flexible open‐shop scheduling problem (FOSP), in which operation sequences are arbitrary and parallel machines can be selected, better matches the actual examination process. Moreover, the chromosome repair mechanism described in Section 5 can enforce item precedence constraints. Therefore, FOSP can more realistically characterize the operational process of clinical health examination centers.

Accordingly, this study abstracts the group health examination scheduling optimization problem as an FOSP. FOSP is an extension of the classical open‐shop scheduling problem (OSP) and the parallel‐machine model and is NP‐hard [1]. To solve related scheduling problems, researchers have designed various metaheuristic algorithms, such as simulated annealing, differential evolution, ant colony optimization, and memetic algorithms [2, 3, 4, 5]. The problem is defined as follows: there are jobs and operations, each operation has several parallel machines, the operation sequence is arbitrary, and some jobs may not require processing in certain operations. At any time, a machine can process only one job, and a job can be processed on only one machine [6]. Once processing of a job starts, interruption is not allowed. The objective is to determine the processing sequence of operations for each job, the assignment of operations to machines, and the processing order of jobs on the assigned machines to optimize certain criteria, such as minimizing completion time.

In recent years, studies on health examination scheduling have mainly aimed to minimize examination duration and have adopted optimization algorithms such as genetic algorithms (GAs) and imperialist competitive algorithms (ICAs). However, as discussed in Section Methods, 2, 6.2, existing studies still have several limitations. First, key real‐world constraints are often missing. Existing studies commonly ignore sex‐related differences, such as female‐specific gynecological examinations and different durations for the same examination item, as well as transition time between examination items. This leads to discrepancies between existing models and the actual operation of health examination centers. Second, existing studies often overlook precedence constraints among some examination items for special populations. Third, most studies address relatively small problem scales, with fewer than 40 examinees and fewer than 6 examination items. Only Zhang et al. [7] explored large‐scale scenarios, but their model was overly simplified, considering only examination duration while ignoring real‐world constraints such as sex differences.

Therefore, this study aims to address the above limitations. First, for general populations, we investigate large‐scale health examination scheduling while considering path‐dependent item transition time, in which travel times between the same two locations may differ by direction, and sex‐related differences in examination items and examination durations. We formulate the batch centralized health examination scheduling problem as an FOSP, construct a scheduling optimization model with the objective of minimizing the maximum examination duration, and solve it using a GA. Second, the model is modified to accommodate precedence constraints among some examination items for special populations, and controlled experiments are designed to verify the effects of introducing constraints on algorithm efficiency and optimization performance. Finally, experimental analyses are conducted, including model verification with small‐scale benchmark examples, case analysis and AnyLogic simulation, and sensitivity analysis, to validate the effectiveness of the model and algorithm and the stability of the solutions.

The main contributions of this study are as follows:

1. We investigate the optimization of batch centralized health examination scheduling in a flexible open‐shop environment for general populations while considering path‐dependent item transition time and sex differences.
2. We establish a mixed‐integer programming (MIP) model with the objective of minimizing the maximum examination duration.
3. We design a GA to solve the model.
4. We modify the model to meet the examination needs of special populations and design controlled experiments to evaluate the effects of introducing constraints on algorithm efficiency and optimization performance.
5. We conduct experimental analyses, including model verification, case analysis and simulation, and sensitivity analysis, to validate the internal and external validity of the model and the stability of the model and algorithm solutions.

The remainder of this paper is organized as follows. Section Methods, 2, 6.2 reviews the current research. Section Results, 3, 6.3 describes the problem and constructs an MIP model that considers path‐dependent item transition time and sex differences. Section Conclusion, 4 presents the GA used to solve the model. Section 5 describes the modified model for precedence constraints among selected examination items in special populations and the controlled experiment used to evaluate the effects of constraint introduction on algorithm performance. Section 6 presents experimental analyses, including model verification, case analysis and simulation, and sensitivity analysis, to validate the effectiveness of the model and the stability of the solutions. Section 7 discusses the innovations, limitations, and future research directions. Finally, Section 8 summarizes the conclusions.

## 2 Literature Review

### 2.1 Health Examination Scheduling Optimization

Health examination scheduling optimization is an important approach for improving medical resource utilization and shortening waiting time. Existing studies have commonly adopted heuristic algorithms such as GAs, with objectives such as minimizing total examination duration and maximizing equipment utilization. One line of research integrates simulation with GAs by using simulation to calculate fitness and optimize health examination scheduling under stochastic service times [8]. However, this method has high computational complexity, and its solution efficiency and stability in large‐scale scenarios require further improvement. Another line of research optimizes processes using preset scheduling rules, such as adjusting examinee sequences by sex and age or dynamically matching examinees according to examination duration and priority [9, 10, 11, 12]. However, these methods rely heavily on rule design, have limited adaptability and flexibility, and are difficult to apply to all constraints in complex real‐world scenarios.

Some studies have also mapped health examination scheduling to complex production scheduling frameworks with medical characteristics, such as FJSP and FOSP. FJSP imposes a fixed sequence on all examination items, whereas FOSP has no sequence restrictions and is more consistent with actual health examination settings. Existing studies have mainly focused on FOSP and designed intelligent algorithms such as GAs and ICAs to shorten total duration and improve examinee satisfaction [7, 13, 14]. Nevertheless, there remains an evident mismatch between these studies and practical scenarios. Some studies considered item transition time but used CPLEX only for small‐scale cases and did not explore the applicability of heuristic algorithms in large‐scale health examination center [13]. Other studies modeled the problem as FJSP or restricted examination sequences to simplify solution procedures, which conflicts with the flexible order of actual examination items, weakens scenario flexibility, and limits the practical implementation of optimization results [14, 15]. In addition, some studies optimized scheduling from the perspective of patient experience. For example, the strategic idleness scheduling strategy proposed by Baron et al. [16] reduces waiting and improves satisfaction by regulating the timing of patient entry into congested stations. However, this strategy depends on complex real‐time monitoring and data analysis and may prolong the total examination duration for other examinees in some scenarios.

Existing research has expanded from examination‐process scheduling optimization to systematic optimization across the entire health examination workflow, covering pre‐examination appointment management, post‐examination specimen testing and report‐writing scheduling, and rescheduling when urgent patients disrupt the examination process. Thus, a full‐process research framework has gradually emerged. However, health examination process scheduling optimization still has deficiencies in scenario adaptability and large‐scale problem solving, and the consistency between theory and actual scenarios needs further improvement [17, 18, 19].

### 2.2 FOSP Research in Healthcare

The FOSP model has substantial application value in healthcare services because it can efficiently match medical resources with patient needs and improve service quality. Patient examination scheduling is highly consistent with FOSP, and related research mainly covers outpatient, inpatient, and emergency department settings.

In outpatient scheduling, early studies designed algorithms with the objective of minimizing total examination or completion time [20], but did not consider patient‐specific differences such as sex, resulting in a mismatch with actual scenarios. Later studies incorporated patient priority and constructed models based on weighted total duration or multi‐objective weighted sums [21, 22]. In scheduling appointments for integrated practice units, Zhang et al. [23] considered both patients and clinic physicians, modeled the care process as an extended OSP, and minimized the weighted sum of clinic closing time and total patient waiting time. However, such studies mainly extended the objective function and still provided limited characterization of real‐world scenarios.

In inpatient scheduling, research has focused on coordinated arrangements of multiple examinations and subsequent medical activities. Paulussen et al. [24] modeled inpatient examination processes as an FOSP and proposed a multi‐agent distributed scheduling method to coordinate resource allocation through a market mechanism [24]. For surgical scheduling, Rahimi et al. [25] modeled the problem as a no‐wait open‐shop surgical case scheduling problem, while Saadani et al. [26] integrated preoperative examinations and surgery into an FOSP with priority constraints to minimize length of hospital stay. However, these models are applicable only to small‐scale scenarios and do not provide heuristic solution schemes for large‐scale problems. These studies demonstrate the applicability of FOSP in complex healthcare processes.

In emergency department scheduling, FOSP is widely used to characterize diagnostic and treatment processes in which patients complete multiple examination items with flexible sequences and parallel resources. Azadeh et al. [27] modeled emergency department examinations as a priority‐based FOSP, minimized weighted waiting time, and used a GA to ensure service quality for high‐priority patients. Other studies divided emergency department processes into two stages: a common preliminary stage and a subsequent flexible examination stage. These studies modeled the problem as a flow‐open shop problem and designed differential evolution algorithms to solve it [28].

In addition, in auxiliary healthcare settings such as pathology laboratories, Azadeh et al. [29] modeled the testing process as an FOSP and proposed a semi‐online GA to address dynamic patient arrivals. Although this work made progress in dynamic scheduling, it did not examine in depth the effect of travel time between examination items.

### 2.3 Research on OSPs

In manufacturing system scheduling, FJSP assumes fixed processing sequences, has a clear solution‐space structure, and has relatively mature modeling methods and solution algorithms. In contrast, FOSP does not restrict operation sequences. Jobs may freely choose processing paths, which increases system flexibility but also expands the solution space and raises computational complexity and solution difficulty. Therefore, related research remains comparatively limited [30]. Gonzalez and Sahni conducted early systematic research on open‐shop scheduling and laid a theoretical foundation. In recent years, FOSP has attracted increasing scholarly attention [31, 32].

To address the computational challenges of high‐dimensional solution spaces, researchers have designed various heuristic and intelligent optimization algorithms for FOSP and its extensions. Matta et al. [33] designed a GA for the proportionate multiprocessor OSP with the objective of minimizing makespan and verified its effectiveness in large‐scale problems. Bai et al. [34] further studied static and dynamic FOSP and used a generalized dense schedule heuristic and differential evolution algorithm to solve large‐ and medium‐scale problems, respectively, thereby improving solution efficiency. Rahmani et al. [35] proposed an extended GA by optimizing crossover and selection operators, improving convergence speed and solution stability. Adak et al. [36] proposed an ant colony optimization algorithm based on implicit stage sequencing for the proportionate FOSP and showed experimentally that it outperformed existing tabu search and scatter search algorithms in solution quality and computational efficiency. However, most of these studies focused on single‐objective makespan minimization and assumed deterministic operation, processing, and transportation times, without fully considering the influence of complex constraints on scheduling results.

In recent years, some studies have begun to focus on multi‐objective optimization and more realistic constraints. Abreu et al. [37] introduced sequence‐dependent setup times into open‐shop scheduling and proposed a hybrid GA with good solution quality and efficiency. Abdelmaguid et al. [38] proposed an exact algorithm based on the constraint method for the dynamic multiprocessor OSP with two objectives. Behnamian et al. [39] constructed a bi‐objective FOSP mixed‐integer nonlinear programming model and designed solution algorithms, verifying the advantages of multi‐objective optimization. Tan et al. [30] further integrated FOSP with automated guided vehicle scheduling and developed a model that minimizes makespan and total carbon emissions, using an enhanced multi‐objective particle swarm optimization algorithm. This expanded the application of FOSP in green manufacturing. However, these studies were mainly based on manufacturing systems and generally assumed fixed transportation times and homogeneous job requirements, making it difficult to reflect the scheduling complexity caused by object heterogeneity in service systems. Although Enayati et al. [40] and Guo et al. [41] introduced transportation time into FOSP, they did not distinguish between different routes or directional travel‐time differences, nor did they model the special requirements of service scenarios such as health examination scheduling, leaving room for improvement.

### 2.4 Simulation Applications in Shop Scheduling

Early studies often used simulation as an analytical and evaluation tool for dispatching rules and system behavior. Kiran et al. [42] used discrete‐event simulation to analyze the effects of dispatching rules and queue‐jumping strategies on system performance under different scenarios [43]. Kim et al. [44] introduced dynamic scheduling rules and evaluated dynamic rule selection through simulation as the production environment changed. Tavakkoli‐Moghaddam et al. [45] developed a job‐shop simulation model that could solve medium‐ and large‐scale problems under specified rules. Lu et al. [46] further explored the combined effects of order review/release control and dispatching rules on system performance. These studies relied on preset rule sets and used simulation to compare the scenario performance of different rules in order to select better solutions. However, they remained at the level of rule evaluation and did not move beyond the rule framework to optimize scheduling schemes themselves.

As research progressed, the focus shifted from rule evaluation to scheduling‐scheme optimization, giving rise to approaches that integrate simulation and optimization algorithms. Yan and Wang [47] used a GA to search for the optimal combination of machines and dispatching rules, and then used simulation to evaluate performance and match appropriate rules to each machine. Gholami et al. [48] considered machine failures and embedded discrete‐event simulation into the GA fitness evaluation process, using performance evaluations under multiple random failure scenarios to guide the search for robust scheduling schemes. Frantzen et al. [49] further investigated job rescheduling after machine failure. Lang et al. [50] introduced an innovative solution method combining deep reinforcement learning with discrete‐event simulation to solve FJSPs, achieving better results than traditional heuristics. These methods can systematically evaluate the performance of scheduling schemes in operating environments that are closer to reality and verify their practical effectiveness.

## 3 Problem Description and Model Construction

### 3.1 Problem Definition and Scope

#### 3.1.1 Problem Description

The process of batch centralized health examinations is as follows. First, the organizational liaison communicates with the hospital or health examination institution in advance to confirm key information such as the number of examinees, the examination date, the selected examination package, whether grouping is needed, and the group list. After this information is confirmed, the organization uniformly arranges buses to transport examinees to the designated examination site at the scheduled time. After arriving at the examination site, examinees collect examination forms in sequence and then proceed to the relevant examination departments in an orderly manner according to the item instructions on the form.

#### 3.1.2 Mapping Between the Batch Health Examination Scheduling Problem and FOSP

FOSP is a classical production scheduling optimization problem characterized by jobs that can be processed on multiple parallel devices and operations that can be performed in arbitrary order. The batch centralized health examination scheduling problem studied in this paper can be mapped one‐to‐one to the FOSP model, as shown in Table 1.

**Table 1:** Mapping between the batch health examination scheduling problem and FOSP.

FOSP element	Examination element	Mapping description
Job	Examinee	Each examinee corresponds to an independent job.
Operation	Examination item	Each examination item, such as internal medicine or surgery, corresponds to one operation.
Parallel machines	Parallel devices for an examination item	Multiple devices configured for the same item, such as multiple ultrasound machines, correspond to parallel machines for an operation.
Processing time	Item examination duration	The time required for an examinee to complete an item on a device corresponds to operation processing time.
Transition time	Inter‐item transition time	The travel time required after one item to reach the next item corresponds to inter‐operation transition time.

#### 3.1.3 Problem Scope and Applicable Scenarios

(1) Problem scope
  Based on actual scenarios, the batch centralized health examination scheduling optimization problem studied in this paper is defined within the following boundaries:
*Object scope*: The study focuses on batch centralized health examination populations and does not include scheduling scenarios for individual examinations. Examinees follow a unified examination package, except for sex‐related differences, and the complex scheduling of personalized customized packages is not considered.
  Resource scope: The number of parallel devices for each examination item is known and fixed. Dynamic adjustments such as equipment failure, temporary additions, or reductions during the examination process are not considered.
*Time scope*: Only on‐site scheduling after examinee arrival is considered. Time optimization for preliminary communication and centralized transportation is not included. Differences in transition time between examination items caused by individual differences are not considered. Variations in examination duration caused by differences in medical staff proficiency or examinee characteristics are also not considered.
(2) Applicable scenarios
  The scheduling optimization model constructed in this study applies to uniformly organized batch centralized health examination scenarios. In this setting, examinees follow a standardized examination package, with only sex‐related item differences and no need for personalized customization. The scheduling mode is non‐real‐time pre‐scheduling; that is, before the scheduled examination day, an optimal examination scheduling scheme is generated in advance based on key information such as population size, selected standardized package, and sex composition, thereby providing forward guidance for efficient on‐site examination workflows.

#### 3.1.4 Modeling Assumptions and Rationale

In FOSP, n examinees need to be assigned to l examination items, and each examination item contains several parallel devices. At any time, one machine can examine only one examinee, and one examinee can undergo only one examination. The sequence of examination items is arbitrary. When calculating the total examination duration, both the duration of each examination item and the transition time between different examination items must be considered. Therefore, for convenience of analysis, based on the studies of Zhang [7] and Yu [14] and the actual operation process of health examination services and the core logic of FOSP, the following assumptions are made:

(1) The duration of each examination item is deterministic and equal across examinees; the examination duration of parallel devices for the same examination item is also equal.

(2) The path‐dependent transition times between different examination items for all examinees are deterministic and known constants.

(3) The distance between parallel devices for the same examination item is ignored.

(4) Once an examination starts, it cannot be interrupted.

(5) Male and female examinees have the same categories of examination items, such as internal medicine, surgery, general examination, blood test, urine test, and ultrasound. Except that female examinees require an additional gynecological examination and that some examination items differ in specific content, such as female ultrasound including an additional gynecological ultrasound and therefore taking longer, there are no other differences.

### 3.2 Mathematical Model Construction

#### 3.2.1 Sets, Parameters, and Variables

The definitions and expressions of the parameters and variables used in the mathematical model are shown in Table 2.

**Table 2:** Definitions of sets, parameters, and decision variables.

Parameters	
n	Total number of batch health examination examinees
l	Total number of examination items
mk	Number of parallel devices for each examination item
M	A sufficiently large positive number
Pk,Gj	Single‐item examination duration for the sex of the examinee on any parallel device of an examination item
tk1,k2	Transition time required to move from one examination item to another
Gj	Binary variable; equals 1 if the examinee is female and 0 otherwise
Sets	
N	Set of examinees, indexed by
L	Set of examination items, indexed by; the gynecological examination is denoted by
Mk	Set of parallel devices for each examination item, indexed by
Variables	
sj,k	Continuous variable indicating the start time of examinee in a department
xj,k,m	Binary variable; equals 1 if examinee is assigned to a parallel device for an examination item and 0 otherwise
yj,k1,k2	Binary variable; equals 1 if an examinee performs one item before another and 0 otherwise
Cj	Continuous variable representing the completion time of an examinee, that is, the ending time of the last completed item
$C_{max}$	Continuous variable representing the maximum examination duration, namely the largest completion time among all examinees

#### 3.2.2 MIP Model

Objective function: min *C*_*max*_

Constraints:

(1) $$\sum_{m\in Mk}xj,k,m=1,\forall j\in N;\forall k\in L\backslash \left\{g\right\},$$

(2) $$\sum_{m\in Mg}xj,g,m=Gj,\forall j\in N,$$

(3) $$\sum_{\begin{array}{l} k\in L\\ k\neq g \end{array}}\sum_{m\in Mk}xj,k,m+Gj\cdot \sum_{m\in Mg}xj,g,m=l-\left(1-Gj\right),\forall j\in N,$$

(4) $$\sum_{j\in N}\sum_{m\in Mk}xj,k,m=n,\forall k\in L\backslash \left\{g\right\},$$

(5) $$\sum_{j\in N}\sum_{m\in Mg}xj,g,m=\sum_{j\in N}Gj,$$

(6) sj2,k≥sj1,k+(xj1,k,m⋅xj2,k,m)⋅pk,Gj1,∀j1,j2∈N;∀k∈L;j1≠j2,

(7) sj1,k≥sj2,k+(xj1,k,m⋅xj2,k,m)⋅pk,Gj2,∀j1,j2∈N;∀k∈L;j1≠j2,

(8) sj,k2≥sj,k1+pk1,Gj+tk1,k2−M(1−yj,k1,k2),∀j∈N,∀k1,k2∈L,k1≠k2,

(9) sj,k1≥sj,k2+pk2,Gj+tk2,k1−M⋅yj,k1,k2,∀j∈N,∀k1,k2∈L,k1≠k2,

(10) Cj=max{(sj,k+pK,Gj)|k∈L},∀j∈N,

(11) $$C_{max}=\;\max\;Cj,\forall j\in N,$$

(12) sj,k≥0,∀j∈N;∀k∈L.

Constraints (1)–(5) ensure that each examinee completes each required examination item once, and that female examinees complete a separate gynecological examination. Constraints (6)–(7) ensure that each examination device serves only one examinee at a time and that device occupation time is consistent with the sex‐specific examination duration of the corresponding examinee. Constraints (8)–(9) ensure that each examinee undergoes only one examination item at a time and that the start times of different items satisfy the requirements of the sex‐specific examination duration and inter‐item transition time. Equation (10) calculates the examination completion time for each examinee, that is, the latest finishing time among all examination items for that examinee. Equation (11) calculates the time at which all examinees complete all examination items, that is, the maximum completion time across all examinees. Constraint (12) ensures that the start time of any examination item for any examinee is non‐negative.

## 4 Solution Algorithm

FOSP is NP‐hard and cannot be solved in polynomial time. Small‐scale problems can be solved optimally using exact algorithms, but the solution time increases exponentially as the problem size grows. Because the large‐scale batch group health examination setting in this study involves many examinees and complex examination items, a GA is used to solve the mathematical model.

### 4.1 Encoding and Initialization

**Figure 1:**
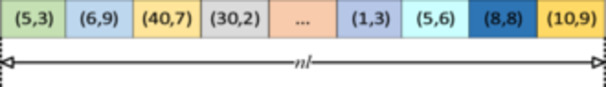
Scheduling plan coding diagram.

The encoding scheme is shown in Figure 1. Each gene is represented as a tuple, with the first position indicating the examinee index and the second position indicating the examination item index. For example, (5,3) indicates that examinee 5 performs examination item 3. For genes corresponding to the same item, the more leftward the gene is positioned in the chromosome, the higher its priority for device assignment. The chromosome contains the mapping relationships between all examinees and all examination items, including invalid mappings in which male examinees go to the gynecological examination. Therefore, the examination duration for male examinees in the gynecological item and the corresponding transition times to and from gynecology are set to 0 in the algorithm, thereby matching the mathematical model constraints and ensuring algorithm validity.

After all mapping relationships between examinees and examination items are generated, the order of these tuples is randomly shuffled to generate the initial population.

### 4.2 Decoding and Fitness

During decoding, chromosome genes are traversed from left to right, and examinees are assigned to the corresponding examination items. Based on the availability time of parallel devices for each item and the arrival time of each examinee, the earliest available device is assigned. The fitness value is then calculated accordingly. Cj

### 4.3 Roulette‐Wheel Selection

Roulette‐wheel selection is a commonly used selection operator in GAs [51]. The probability that an individual is selected is positively associated with its fitness, and elitist retention can be used to transmit high‐quality genes.

### 4.4 Crossover Operation

Crossover refers to the process of randomly selecting two individuals from the population as parents and combining chromosome segments from the two parents with a given probability and crossover operator to generate offspring [52]. The crossover operator used in this study is position‐based crossover (PBX).

**Figure 2:**
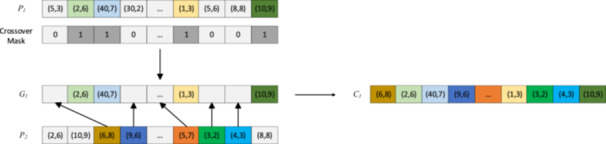
Position‐based crossover operator.

Figure 2 shows the operation process of this crossover operator. (1) A crossover mask consisting of nl random integers of 0 or 1 is generated, where genes with a value of 1 participate in the crossover operation. (2) Two parent individuals, denoted as *P* 1 and *P* 2, are selected from the current population. (3) Genes with mask values of 1 are extracted from *P* 1 and arranged in their original order to form a new gene segment *G* 1; genes already contained in *G* 1 are eliminated from *P* 2, and the remaining genes are sequentially filled into *G* 1 to generate the new offspring *C* 1.

### 4.5 Mutation Operation

**Figure 3:**
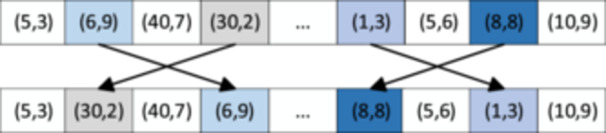
Mutation operation.

Mutation refers to the process of applying a mutation operator to offspring with a certain probability, which helps increase population diversity. To ensure chromosome legality, this study uses the swap mutation operator [53], in which genes at two randomly selected positions are exchanged, as shown in Figure 3.

## 5 Model Improvement

Special group health examinations, such as those for military personnel, may involve precedence constraints among examination items, which are not incorporated in the preceding model. To improve model generalizability and cover more types of group health examinations, this study modifies the model and algorithm to accommodate the examination needs of such special populations.

### 5.1 Modification of the Mathematical Model

**Additional set:**

A={(k1,k2)∈L×L∣k1≠k2}: the set of ordered pairs of examination items with precedence constraints, where the ordered pair (k1,k2) indicates that all examinees must complete item k1 before item k2.

**Additional constraint:**

(13) sj,k2≥sj,k1+pk1,G+tk1,k2,∀j∈N,∀(k1,k2)∈A,k1≠k2.

Constraint (13) ensures that examination items with precedence constraints are correctly implemented while satisfying sex‐specific examination duration requirements.

### 5.2 Modification of the GA

A repair procedure is added after the encoding and initialization, crossover, and mutation operations of the original GA to ensure that generated chromosomes satisfy examination‐item precedence constraints. All other components remain unchanged. The pseudocode is shown in Table 3.

**Table 3:** Pseudocode of the chromosome correction algorithm.

Algorithm 1: Chromosome correction algorithm		
	**Input:**	Constraint matrix composed of ordered pairs of examination items with sequential constraints A, Sample chromosome sampleChromeQ
	**Output:**	Corrected chromosome sampleChromeQ. 2
**1**	sampleChormeQ. 2 ← sampleChromeQ;	
**2**	soldierQty ← max(sampleChromeQ(:, 1));	
**3**	procQty ← max(sampleChromeQ(:, 2));	
**4**	constrained ← unique(concat(A(:, 1), A(:, 2)));	
**5**	adj_list ← cell(procQty, 1);	
**6**	**for** i ← 1 **to** size(A, 1) **do**	
**7**	u ← A(i, 1), v ← A(i, 2);	
**8**	**if** u ≤ procQty **then**	
**9**	adj_list{u} ← [adj_list{u}, v];	
**10**	**end**	
**11**	**end**	
**12**	visited ← zeros(1, procQty), order ← [];	
**13**	nextIdx ← ones(1, procQty);	
**14**	**foreach** node ∈ constrained **do**	
**15**	**if** visited(node) ≠ 0 **then**	
**16**	**end**	
**17**	stack ← [node], visited(node) ← 1;	
**18**	**while** stack ≠ ∅ **do**	
**19**	current ← stack(end);	
**20**	**if** nextIdx(current) ≤ len(adj_list{current}) **then**	
**21**	next ← adj_list{current}(nextIdx(current));	
**22**	nextIdx(current) ← nextIdx(current) + 1;	
**23**	**if** visited(next) = 0 **then**	
**24**	visited(next) ← 1, stack ← [stack, next];	
**25**	**end**	
**26**	**else**	
**27**	visited(current) ← 2, order ← [current, order];	
**28**	removecurrentfromstack;	
**29**	**end**	
**30**	**end**	
**31**	**end**	
**32**	**for** s ← 1 **to** soldierQty **do**	
**33**	rows ← f ind(sampleChromeQ. 2(:, 1) = s);	
**34**	constrainedRows ← f ilter(rows, sampleChromeQ. 2(:, 2) ∈ constrained);	
**35**	procIDs ← sampleChromeQ. 2(constrainedRows, 2);	
**36**	[, pos] ← index(procIDs, order);	
**37**	[, sortedIdx] ← sort(pos);	
**38**	newP rocIDs ← procIDs(sortedIdx);	
**39**	sampleChromeQ. 2(constrainedRows, 2) ← newProcIDs;	
**40**	**end**	
**41**	**return** sampleChromeQ. 2;	

#### 5.2.1 Repair Mechanism

**Figure 4:**
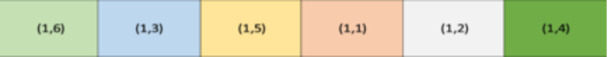
Initial chromosome.

The chromosome repair mechanism satisfies constraints by adjusting the positions of selected genes. Taking the six examination items of examinee 1, numbered 1–6, as an example, the initial chromosome in Figure 4 corresponds to the examination sequence 6 → 3 → 5 → 1 → 2 → 4.

**Figure 5:**
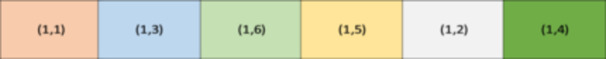
Corrected chromosome.

Suppose two precedence constraints are added: examination item 1 must precede item 6, and item 3 must precede item 5. During repair, the constrained items [1, 3, 5, 6] are first extracted, and a feasible order, such as [1, 3, 5, 6], is obtained through topological sorting. This sequence is then replaced in situ at the corresponding gene positions of the constrained items in the initial chromosome, namely positions 1, 2, 3, and 4, to obtain the corrected chromosome shown in Figure 5.

### 5.3 Computational Burden Analysis of the Chromosome Repair Mechanism

To evaluate the effect of introducing examination‐item precedence constraints on the solution performance of the model and algorithm, this study designed a controlled experiment. The analysis was conducted from two dimensions, computation time and scheduling performance, to quantitatively assess the effect of the chromosome repair mechanism on algorithm efficiency and clarify its practical application value.

#### 5.3.1 Experimental Design

(1) Basic experimental setting

Using the case in Section Methods, 2, 6.2 as the background, a small‐scale test instance was constructed. The instance included 30 examinees, comprising 27 men and 3 women, and 12 examination items. Resource configuration, such as the number of devices, item transition times, and GA parameters, including population size and crossover and mutation probabilities, were kept consistent with Section Methods, 2, 6.2.

(2) Constraint‐gradient setting

To systematically examine the effects of the number of constraints on algorithm performance, four experimental groups with different constraint intensities were randomly generated based on common precedence constraints in actual health examinations, as shown in Table 4.

**Table 4:** Details of the four experimental groups.

Group	Number of examinees	Number of examination items	Number of constraint sets	Constraint content
Unconstrained group	30	12	0	None
Constraint group A	30	12	1	R1
Constraint group B	30	12	2	R1, R2
Constraint group C	30	12	3	R1, R2, R3

*Note:* R1: Ultrasound before general examination; R2: ECG before X‐ray; R3: Blood test before psychological assessment.

(3) Evaluation indicators

*Computation time*: Each group was independently run 10 times. The total solution time of the algorithm was recorded, and the average value (Avg), best value (Best), and standard deviation (St Dev) were calculated to reflect the influence of the repair mechanism on solution efficiency.

*Scheduling scheme*: Each group was independently run 10 times. The maximum examination duration (*C_max_*) was used as the indicator, and Avg, Best, and St Dev were calculated for each group to evaluate the effect of constraint introduction on optimization performance.

#### 5.3.2 Solution Results and Analysis

1. Computation‐time analysis

The computation‐time statistics for each group are shown below.

As shown in Table 5, after introducing the repair mechanism, the best computation times of the constraint groups were slightly higher than that of the unconstrained group. However, the average times showed no clear pattern, and the increases were all within 20 s, indicating that the repair mechanism had only a minor effect on algorithm efficiency. Meanwhile, the standard deviations of computation time in the constraint groups were lower than that in the unconstrained group, suggesting that the constraints narrowed the solution space, reduced solution variability, and improved time stability.

**Table 5:** Comparison of computation time and solution results across groups.

Evaluation indicator/group	Computation time (s)			Scheduling scheme (min)		
	Avg	Best	St Dev	Avg	Best	St Dev
Unconstrained group	391.69	354.43	35.48	74.96	73.69	0.82
Constraint group A	397.56	376.77	22.95	84.48	82.69	1.16
Constraint group B	389.92	372.24	25.29	101.64	98.04	1.76
Constraint group C	410.60	388.23	29.08	108.50	104.65	1.79

Regarding scheduling performance *C_max_*, both the best and average values increased as the number of constraints increased. This indicates that precedence constraints reduce the optimization space while ensuring the rationality of the physical examination process, resulting in an increase in *C_max_*. However, the standard deviations of *C_max_* for all constraint groups were low across all groups, indicating that the repair mechanism can stably generate feasible solutions satisfying the constraints and does not lead to a loss of solution quality due to the introduction of constraints.

In summary, the repair mechanism introduces only a slight efficiency loss while ensuring feasible solutions and can meet the practical need for multi‐constraint processing in health examination scheduling.

## 6 Experimental Analysis

To systematically evaluate the reliability of the proposed model and algorithm, this study conducted empirical analyses from three perspectives: model verification, simulation, and sensitivity analysis. First, model verification was used to ensure the correctness of the internal algorithm implementation. Next, a real case and independent simulation were used for external validation to test the model's effectiveness in scenarios approximating real operations. Finally, sensitivity analysis was used to evaluate the stability of the solution. The case analysis directly demonstrated the optimization performance of the model and algorithm. All experimental analyses were performed on a Lenovo computer with an 11th Gen Intel(R) Core(TM) i5‐1155G7 processor, 2.50 GHz, and 16.0 GB RAM.

### 6.1 Model Verification: Small‐Scale Benchmark Examples

To verify the internal logical consistency of the mathematical model and the basic rationality of algorithmic scheduling, this study used model verification. Because the model integrates real‐world constraints such as sex differences and path dependence, the existing literature lacks relevant benchmark instances. Therefore, based on the case background in Section Methods, 2, 6.2, two extremely simplified instances were constructed to verify the internal logic of the model and algorithm.

The instances were designed as follows. Each instance included only one examinee, all equipment resources were set to one unit, and six examination items were randomly selected, with male examinees not undergoing gynecological examination. In addition, the purpose of this solution was to verify the basic validity of the model rather than to pursue optimal scheduling performance under complex scenarios. Therefore, the number of GA iterations was set to 1, while all other parameters were kept consistent with Section Methods, 2, 6.2. The detailed settings are shown in Table 6. MATLAB R2024b was used for solution, and the scheduling results are shown in Tables 7 and 8.

**Table 6:** Details of the test instances.

Instance	Sex	Examination item
Instance 1	Female	ECG, blood test, internal medicine, surgery, gynecology, ultrasound
Instance 2	Male	ECG, blood test, internal medicine, surgery, ultrasound

**Table 7:** Scheduling results for instance 1.

Examination item	Start time	End time	Examination‐duration difference (min)	Transfer‐time difference (min)
ECG	0	3	0	0
Gynecology	3.16	6.16	0	0
Ultrasound	6.29	17.66	0	0
Surgery	17.76	18.96	0	0
Internal medicine	19.01	20.26	0	0
Blood test	20.57	21.37	0	0

**Table 8:** Scheduling results for instance 2.

Examination item	Start time	End time	Examination‐duration difference (min)	Transfer‐time difference (min)
ECG	0	3	0	0
Ultrasound	3.2	10.52	0	0
Surgery	10.62	11.82	0	0
Internal medicine	11.87	13.12	0	0
Blood test	13.43	14.23	0	0
Gynecology	—	—	—	—

*Note:* “—” indicates no scheduling result.

In addition, to verify the effectiveness of the chromosome repair mechanism in Section 5, a precedence constraint requiring internal medicine to precede ultrasound was added to the two instances above, while all other settings remained unchanged. MATLAB R2024b was used for solution, and the results are shown in Table 9.

**Table 9:** Solution results after adding the precedence constraint.

Instance	Scheduling scheme (ordered from left to right)
Instance 1	Blood test, internal medicine, ECG, gynecology, ultrasound, surgery
Instance 2	Blood test, internal medicine, surgery, ultrasound, ECG

#### 6.1.1 Result Analysis

Tables 7 and 8 present the examination sequences planned by the model for a single examinee from top to bottom. To quantify the verification results, this study defined two indicators:

*Examination‐duration difference*: The difference between the ending time and starting time for each item in the table minus its actual examination duration, as shown in Table 10 of Section Methods, 2, 6.2.

**Table 10:** Correspondence between examination items and parallel device IDs.

US	ECG	Blood	Int Med	Surg	GEN	ENT	X‐ray	Ophth	Urine	Psych	Gyn
1–4	5–6	7–9	10	11	12	13	14	15	16–25	26–45	46

Abbreviations: Gen, general examination; Gyn, gynecology; Int Med, interal medicine; Ophth, ophthalmology; Psych, psychological assessment; Surg, surgery; US, ultrasound.

*Transfer‐time difference*: The difference between the starting time of an adjacent item and the ending time of the previous item minus the corresponding actual transfer time, as shown in Table 11 of Section Methods, 2, 6.2.

**Table 11:** Inter‐item transition‐time matrix (min).

US	ECG	Blood	Int Med	Surg	GEN	ENT	X‐ray	Ophth	Urine	Psych	Gyn
0	0.20	0.47	0.16	0.10	0.47	0.14	0.37	0.17	0.13	0.78	0.13
0.20	0	0.44	0.13	0.14	0.44	0.10	0.22	0.05	0.10	0.76	0.16
0.50	0.47	0	0.34	0.39	0.00	0.51	0.09	0.48	0.09	0.31	0.54
0.16	0.13	0.31	0	0.05	0.31	0.17	0.22	0.14	0.20	0.62	0.20
0.10	0.14	0.36	0.05	0	0.36	0.14	0.27	0.13	0.17	0.67	0.17
0.50	0.47	0.00	0.34	0.39	0	0.51	0.09	0.48	0.09	0.31	0.54
0.14	0.10	0.48	0.17	0.14	0.48	0	0.38	0.05	0.05	0.79	0.05
0.40	0.25	0.09	0.25	0.30	0.09	0.41	0	0.39	0.19	0.40	0.45
0.17	0.05	0.45	0.14	0.13	0.45	0.05	0.36	0	0.10	0.76	0.10
0.13	0.10	0.09	0.20	0.17	0.09	0.05	0.19	0.10	0	0.25	0.09
0.84	0.82	0.34	0.68	0.73	0.34	0.85	0.43	0.82	0.25	0	1.08

Abbreviations: Gen, general examination; Gyn, gynecology; Int Med, interal medicine; Ophth, ophthalmology; Psych, psychological assessment; Surg, surgery; US, ultrasound.

As shown in Tables 7 and 8, both indicators were 0. The ultrasound durations in the two instances were consistent with the actual values, 7.32 min for male examinees and 11.37 min for female examinees. Table 8 also shows that instance 2 did not include the gynecological examination. In addition, the solution results in Table 9 satisfied the precedence constraint requiring internal medicine to precede ultrasound. These findings indicate that the model accurately calls the parameters for examination duration and transfer time and correctly calculates time. They also confirm that the model's basic scheduling logic is consistent with the predefined rules under constraints such as sex differences, path dependence, and precedence relationships among examination items, thereby validating the model.

### 6.2 Case Analysis and Simulation Verification

To verify the external validity of the model and algorithm, this study selected a large‐scale batch health examination event at a hospital health examination center as an empirical case and conducted field investigation. Basic data, including the number and sex of examinees, examination package type, maximum examination duration, and medical resource configuration, were collected from the internal operating records of the health examination center. For each examination item, 5–6 examinees were randomly selected for field measurement, and item examination durations and transfer times between examination items were recorded. The mean values were used as quantitative inputs for the above indicators. Because the parameters were obtained from small‐sample field measurements, no complete descriptive statistics or statistical inference analyses were conducted for the relevant variables. These values were used only as input parameters for the scheduling model to represent the average operating level of the examination process.

#### 6.2.1 Case Background

A batch examination at a hospital health examination center involving 100 general examinees from a non‐special population was selected. The maximum examination duration was 220 min, and the male‐to‐female ratio was 10:1, including 91 men and 9 women. Data on item‐specific examination durations, hospital resource configuration, transition times between examination items, and sex ratio were used as input parameters for the GA model to solve the health examination scheduling scheme. Detailed parameters are shown in Tables 10, 11, 12.

**Table 12:** Resource configuration and examination duration of each item.

Examination item	Number of rooms/devices	Examination time (min)
Ultrasound	4	Female: 11.37 Male: 7.32
ECG	2	3.00
Blood test	3	0.80
Internal medicine	1	1.25
Surgery	1	1.20
General examination	1	1.40
ENT	1	1.00
X‐ray	1	1.00
Ophthalmology	1	1.30
Urine test	10	1.20
Psychological assessment	20	7.00
Gynecology	1	3.00

#### 6.2.2 Solution Results

**Figure 6:**
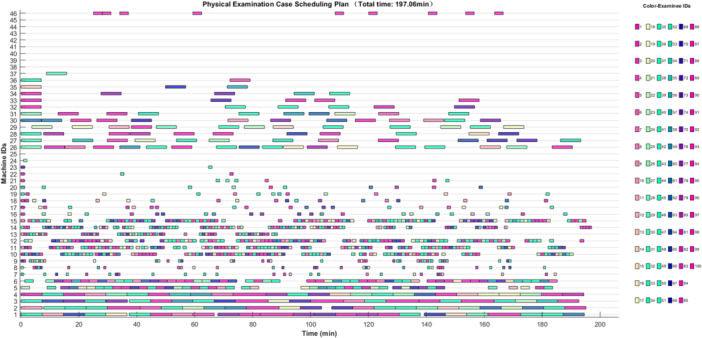
Gantt chart of the best scheduling scheme in the health examination case.

After the above parameters were input into the GA model, the population size was set to 100, the crossover probability to 0.7, the mutation probability to 0.05, and the maximum number of iterations to 200. The algorithm was independently run 10 times. The mean solution value was 198.18 min, the best value was 197.06 min, and the standard deviation was 0.75. Because better scheduling results are preferred in practice, the scheduling scheme corresponding to the best value was used in the subsequent analysis. The optimal scheduling Gantt chart for this case is shown in Figure 6.

The horizontal axis represents time (min), and the vertical axis represents examination device IDs. Each rectangular bar represents one examinee, and different colors distinguish examinees, as shown in the legend on the right. The length of each bar represents examination duration and visually reflects the time arrangement and equipment utilization in the examination process.

#### 6.2.3 Simulation Verification

Practical evidence shows that simulation can fully account for variable random factors such as patient arrival and resource status and is more flexible in evaluating the effects of healthcare system interventions. Therefore, it has been widely applied in medical modeling [54]. Studies have also shown that approximately 32% of studies related to healthcare services and appointment scheduling use AnyLogic for simulation modeling [55]. Accordingly, this study used AnyLogic 8.9.0 Professional to construct a discrete‐event simulation model for the above case to verify the applicability of the obtained scheduling scheme in practical settings.

A health examination simulation model was constructed based on the process library of AnyLogic to digitally simulate the real health examination scenario. The model input parameters included item‐specific examination durations, hospital resource configuration, transition times between examination items, sex ratio, and a time unit of minutes. The examination path of examinees in the model was set as follows: 100 examinees were injected at one time into the source module using the inject function; examinees underwent examinations according to the scheduling scheme; and 100 simulation runs were performed to test the stability of the simulation results for the optimized scheme. The results showed that the simulated total examination duration was 195.57 min. Compared with the GA‐calculated value of 197.06 min, the error was 1.49 min.

#### 6.2.4 Result Analysis

The maximum examination duration of the GA‐optimized scheduling scheme was 197.06 min, and the AnyLogic simulation result was 195.57 min, with an error of 1.49 min. Compared with the pre‐optimization duration of 220 min, the simulation result was shortened by 24.43 min, corresponding to an optimization rate of 11.10% (24.43 min/220 min). These findings validate the effectiveness of the model and algorithm solutions and provide quantitative support for addressing large instantaneous flows and disorder in batch centralized health examinations.

The 1.49‐min discrepancy mainly arose from differences between the algorithm output and the simulation settings. The GA‐generated scheduling scheme included the specific service order of examinees on each device, whereas actual health examination centers generally do not impose a strict service sequence within a single device. Therefore, the simulation constrained only the order of examination items and did not restrict the within‐device service sequence.

In addition, this simulation was deterministic. On the one hand, the inputs were fixed, as all input parameters were fixed measured values. On the other hand, the simulated examination process was also deterministic to a certain extent. Although the simulation model did not specify the service sequence of examinees on each device, the examination items and device assignments of the 100 examinees had been predefined. Therefore, all 100 repeated simulation runs yielded the same result of 195.57 min, with no data fluctuation. This confirmed the stability of the simulation results for the optimized scheme; therefore, statistics such as the mean and standard deviation were not reported.

Further analysis of Figure 6 showed idle devices in psychological examination (machines 38–45) and urine testing (machine 25). This phenomenon may be attributable to the GA device‐allocation strategy. During task assignment, the algorithm searches the parallel device IDs corresponding to each examination item in ascending order until it identifies the earliest available device. In other words, when earlier‐numbered devices can meet task requirements, the algorithm does not continue searching later devices, resulting in some devices not being used.

### 6.3 Sensitivity Analysis

**Figure 7:**
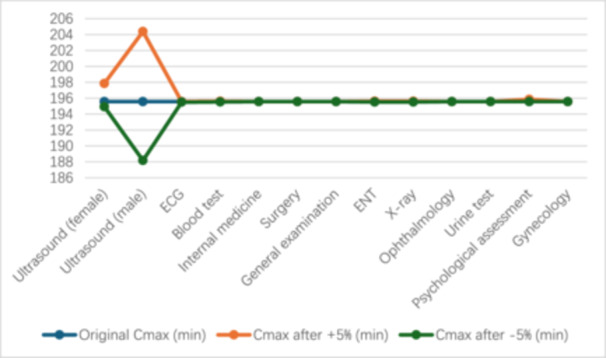
Sensitivity analysis results after ±5% variation in examination duration of each item.

**Figure 8:**
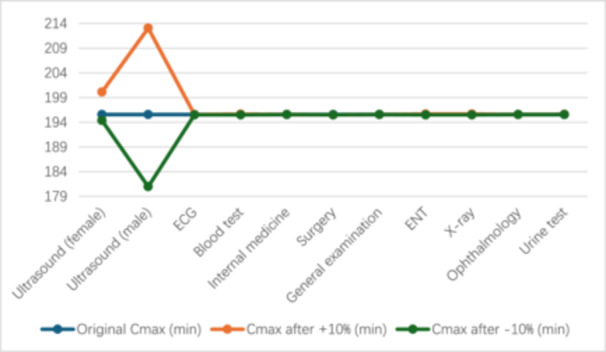
Sensitivity analysis results after ±10% variation in examination duration of each item.

To verify the stability of the model and algorithm solutions, sensitivity analysis was conducted based on the case and optimized scheme in Section Methods, 2, 6.2. With all other conditions unchanged, the service durations of each examination item in the simulation model for the optimized scheme described in Section 2.3, 6.2.3 were varied within ±5% and ±10% to examine their effects on the total examination duration of *C_max_* 195.57 min. The results are shown in Figures 7 and 8. As noted in Section 2.4, 6.2.4, the simulation in this study was deterministic, and repeated runs did not generate random fluctuations. Therefore, the figures present only fixed simulation outputs, without reporting means or standard deviations.

#### 6.3.1 Result Analysis

The total examination duration is defined as the total time required for the last examinee in the system to complete all items. Whether this indicator changes depends on whether the variation in a single item's duration affects the examination process of the last examinee. As shown in Figure 6, the ultrasound Gantt bars are arranged very densely, indicating low equipment idle time and nearly zero available slack [56].

Combined with the sensitivity results in Figures 7 and 8, ultrasound was the most sensitive component influencing total examination duration. Male ultrasound showed the highest sensitivity, as both ±5% and ±10% bidirectional variations in duration caused substantial changes in total examination duration. Female ultrasound significantly prolonged overall progress only when the examination duration increased, whereas shortening the duration had almost no effect on total duration. Small variations of ±5% and ±10% in items such as ECG, blood sampling, imaging, internal medicine, and surgery were fully absorbed by the system and did not change the total examination duration. Comparison of Figures 7 and 8 indicates that larger disturbances in single‐item duration expanded the fluctuation range of total duration caused by the ultrasound bottleneck.

Overall, the solution generated by the proposed model showed good stability under reasonable disturbances of ±5% and ±10% in examination duration. Small duration deviations in most departments did not interfere with the overall examination progress. In practical operations, the service rhythm of the ultrasound department should be carefully managed, and ultrasound examination duration should be stabilized to reduce the constraint imposed by this bottleneck on overall examination efficiency.

## 7 Discussion

Compared with previous studies, the innovation of this study lies in its full consideration of practical constraints in health examination scheduling, including path‐dependent transition time between examination items, sex differences, and precedence constraints among some examination items. Many previous studies, such as those by Zhang [7] and Liu [8], ignored these constraints, leading to models that deviate from reality. Other studies developed relatively general models but did not consider sex differences, path dependence, and other factors, resulting in weak adaptability to actual health examination scheduling scenarios [40, 41]. Although Wang [13] and Yu et al. [14] considered transition time between items, the former did not explore intelligent algorithms such as GAs for large‐scale scenarios, and the latter modeled the problem as FJSP with mandatory fixed examination sequences, which is inconsistent with the actual arbitrary order of examination items.

This study constructed a health examination scheduling optimization model that considers path‐dependent item transition time and sex differences, modified the model for special populations with precedence constraints among selected examination items, and finally verified its effectiveness and stability through experimental analyses. The proposed model provides a scientific scheduling decision‐making basis for hospitals and health examination institutions, helps improve examinee experience, alleviates pressure on medical resources, and positively contributes to improving the operational management and service quality of batch centralized health examinations. In current healthcare service environments, such as outpatient departments and health examination centers, process optimization often relies on artificial intelligence, big data, positioning, and sensor devices to provide intelligent patient guidance and queue management. Relevant practices have improved operational efficiency and patient experience to some extent, but they usually require additional hardware deployment, multi‐system interface integration, and substantial process reengineering [57, 58]. In contrast, the open‐shop scheduling optimization framework proposed in this paper can be modeled and solved using only existing examination‐item and resource information in the health examination process, without additional hardware investment or system restructuring. While ensuring optimization effectiveness, it is therefore more feasible and scalable for implementation.

Although the proposed model and method achieved favorable optimization effects in the batch centralized health examination scenario, several limitations remain. First, deterministic assumptions were adopted in the modeling and simulation processes. Service durations of examination items and transition times between items were treated as known constants, and random fluctuations and uncertainties such as stochastic arrivals and individual differences in examination duration were not considered. Second, although the sensitivity analysis of service‐duration fluctuations verified the stability of scheduling results within a certain range, unexpected events such as equipment failure and temporary queue jumping were not further considered. The adaptability of the scheduling scheme in complex stochastic environments therefore requires further investigation. Third, external validation of the model and algorithm mainly relied on a single case with a male‐to‐female ratio of 10:1. Multi‐scenario validation under different sex structures, examination scales, and resource configurations was lacking, and the generalizability of the model to different examination institutions or spatial layouts requires further testing.

Future research can proceed in several directions. First, uncertainties such as equipment failure and temporary queue jumping can be incorporated into health examination scheduling models. Second, comparative experiments across multiple cases, institutions, or layout conditions can be conducted to systematically evaluate the external validity of the model and algorithm. Third, multisource real‐world operational data can be integrated to further verify the applicability and scalability of the proposed method in more complex and dynamic health examination environments.

In reporting statistical methods, this study followed the statistical reporting standards proposed by Assel et al. [59] to ensure transparency and consistency of experimental results.

## 8 Conclusions

This study addressed the problems of numerous examination items, complex routes, and easy congestion in batch centralized health examinations by constructing a flexible open‐shop health examination scheduling optimization model that considers path‐dependent item transition time and sex differences. A GA was used to solve the model, and the model was extended to support special‐population scenarios with precedence constraints among selected examination items. The experimental results showed that the internal operating logic of the model and algorithm was reasonable and that the model could generate solutions according to predefined scheduling rules. In the real case, the optimized maximum examination duration was 197.06 min, and the AnyLogic simulation result was 195.57 min. Compared with the pre‐optimization duration of 220 min, the duration was shortened by 24.43 min, improving overall efficiency by 11.10% (24.43 min/220 min). The theoretical and simulation results were in good agreement, reflecting the practical applicability of the model to some extent. Sensitivity analysis showed that, within a reasonable range of parameter fluctuation, the system could effectively buffer duration changes for most examination items, indicating that the resulting scheduling scheme had a certain degree of stability.

Overall, the proposed model and algorithm are expected to improve the operational efficiency of batch centralized health examinations without additional hardware investment and may provide useful reference value.

## Author Contributions

Conceptualization: Xin Wang and Yang Kong. Visualization: Xiaoming Zhao and Xuchen Pan. Funding Acquisition: Xin Wang. Writing and editing: Mengdi Lv and Xiaoming Zhao. Writing–original draft: Xin Wang and Yang Kong.

## Disclosure

All authors have read and approved the final version of the manuscript. Xin Wang and Yang Kong had full access to all of the data in this study and take complete responsibility for the integrity of the data and the accuracy of the data analysis. The funding sources had no involvement in study design, collection, analysis, and interpretation of data, writing of the report, or the decision to submit the report for publication.

## Ethics Statement

This study was approved by the Institutional Ethics Committee of the 970 Hospital of the PLA Joint Logistic Support Force (Ethics Approval No. 2025‐NO.12/WQC). The whole research strictly complies with the ethical principles outlined in the Declaration of Helsinki, and the privacy and personal rights of all subjects are fully protected.

## Consent

All participants were fully informed of the research purpose, procedures, potential risks, and benefits before voluntary signing written informed consent to participate in this study. No patients were involved in this study. The public were not involved in the study design/conduct, the data analysis/interpretation or in the preparation of the manuscript.

## Conflicts of Interest

The authors declare no conflicts of interest.

## Transparency Statement

Xin Wang and Yang Kong affirm that this manuscript is an honest, accurate, and transparent account of the study being reported; that no important aspects of the study have been omitted; and that any discrepancies from the study as planned (and, if relevant, registered) have been explained.

## Data Availability

Data sharing is not applicable to this article as no new data were created or analyzed in this study.

## References

[1] W. Mao , “Multi‐Operation Multi‐Machine Scheduling.” High‐Performance Computing and Networking: International Conference and Exhibition, Milan, Italy, May 3‐5 (Springer, 1995). 1995, 33–38.

[2] S. H. Shams Lahroudi , F. Mahalleh , and S. Mirkamali , “Multiobjective Parallel Algorithms for Solving Biobjective Open Shop Scheduling Problem,” Complexity 2022 (2022): 5043058.

[3] L. He , Y. Cao , W. Li , J. Cao , and L. Zhong , “Optimization of Energy‐Efficient Open Shop Scheduling With an Adaptive Multi‐Objective Differential Evolution Algorithm,” Applied Soft Computing 118 (2022): 108459.

[4] M. Kurdi , “Ant Colony Optimization With a New Exploratory Heuristic Information Approach for Open Shop Scheduling Problem,” Knowledge‐Based Systems 242 (2022): 108323.

[5] I. K. Mashizi , V. M. Kermani , and N. Shahsavari‐Pour , “Flexible Open‐Shop Problem for Minimizing Weighted Total Completion Time,” Journal of Intelligent & Fuzzy Systems 42, no. 3 (2022): 1353–1366.

[6] Z. Adak , M. Ö. Arıoğlu Akan , and S. Bulkan , “Multiprocessor Open Shop Problem: Literature Review and Future Directions,” Journal of Combinatorial Optimization 40 (2020): 547–569.

[7] J. Zhang , L. Wang , and L. Xing , “Large‐Scale Medical Examination Scheduling Technology Based on Intelligent Optimization,” Journal of Combinatorial Optimization 37 (2019): 385–404.

[8] D. Liu and N. Geng , “Stochastic Health Examination Scheduling Problem Based on Genetic Algorithm and Simulation Optimization.” 2020 IEEE 7th International Conference on Industrial Engineering and Applications (ICIEA (IEEE, 2020), 620–624.

[9] C. C. Chern , P. S. Chien , and S. Y. Chen , “A Heuristic Algorithm for the Hospital Health Examination Scheduling Problem,” European Journal of Operational Research 186, no. 3 (2008): 1137–1157.

[10] R. K. Chiu and Y. C. Yeh , “Fuzzy‐Based Dynamic Scheduling System for Health Examination.” 2010 International Conference on Machine Learning and Cybernetics (IEEE, 2010). 2, 636–641.

[11] X. Hu , H. Wu , S. Zhang , et al., “Scheduling Outpatients in Hospital Examination Departments.” 2009 IEEE International Conference on Industrial Engineering and Engineering Management (IEEE, 2009), 335–338.

[12] M. Ito , S. Hara , M. Tanaka , and R. Takashima , “Examination‐Order Scheduling for Minimizing Waiting Time: A Case Study of a Medical Checkup,” Operations Research for Health Care 22 (2019): 100190.

[13] H. M. Wang and F. D. Chou , “A Mixed Integer Programming Method for the Health Examination Center Scheduling Problems With Sequence‐Dependent Transportation Time,” Journal of Advanced Management Science 4, no. 5 (2016): 426–429.

[14] H. Yu , J. Li , L. Zhang , and P. Duan , “An Imperialist Competition Algorithm Using a Global Search Strategy for Physical Examination Scheduling,” Applied Intelligence 51 (2021): 3936–3951. 34764571 10.1007/s10489-020-01975-y PMC7680665

[15] A. Ala , S. E. Torkayesh , A. E. Torkayesh , and A. Iranizad , “A Hybrid Genetic Algorithm for Appointment Scheduling in a Health Examination System,” International Journal of Value Chain Management 11, no. 4 (2020): 293–310.

[16] O. Baron , O. Berman , D. Krass , et al., “Strategic Idleness and Dynamic Scheduling in an Open‐Shop Service Network: Case Study and Analysis,” Manufacturing & Service Operations Management 19, no. 1 (2017): 52–71.

[17] T. W. Ho , L. C. Kung , H. Y. Huang , J. F. Lai , and H. M. Chiu , “Overbooking for Physical Examination Considering Late Cancellation and Set‐Resource Relationship,” BMC Health Services Research 21 (2021): 1254. 34801021 10.1186/s12913-021-07148-y PMC8605579

[18] Y. Wang , B. Fan , J. Zhai , and W. Xiong , “Two‐Machine Flowshop Scheduling in a Physical Examination Center,” Journal of Combinatorial Optimization 37 (2019): 363–374.

[19] D. D. Zhu , J. Q. Sun , and Y. Zhao , “A Hybrid GA‐SA for the Urgent Patients Disturbed Physical Examination Rescheduling Problem Considering Setup Time,” IEEE Access 9 (2021): 14787–14806.

[20] M. E. Matta and S. E. Elmaghraby , “Polynomial Time Algorithms for Two Special Classes of the Proportionate Multiprocessor Open Shop,” European Journal of Operational Research 201, no. 3 (2010): 720–728.

[21] Z. Abtahi , R. Sahraeian , and D. Rahmani , “A Stochastic Model for Prioritized Outpatient Scheduling in a Radiology Center,” International Journal of Engineering Transactions A: Basics 33, no. 4 (2020): 598–606.

[22] T. F. Abdelmaguid , “Bi‐Objective, Dynamic, Multiprocessor Open‐Shop Scheduling: A Hybrid Scatter Search‐Tabu Search Approach,” Algorithms 17, no. 8 (2024): 371.

[23] P. Zhang , J. F. Bard , D. J. Morrice , and K. M. Koenig , “Extended Open Shop Scheduling With Resource Constraints: Appointment Scheduling for Integrated Practice Units,” IISE Transactions 51, no. 10 (2019): 1037–1060.

[24] T. O. Paulussen , N. R. Jennings , K. S. Decker , and A. Heinzl , “Distributed Patient Scheduling in Hospitals.” Proceedings of the 18th International Joint Conference on Artificial Intelligence (IJCAI'03) (Morgan Kaufmann Publishers Inc, 2003), 1224–1229.

[25] A. Rahimi , S. M. Hejazi , M. Zandieh , and M. Mirmozaffari , “A Novel Hybrid Simulated Annealing for No‐Wait Open‐Shop Surgical Case Scheduling Problems,” Applied System Innovation 6, no. 1 (2023): 15.

[26] N. E. H. Saadani , Z. Bahroun , and A. Bouras , “A Linear Mathematical Model for Patients' Activities Scheduling on Hospital Resources.” 2014 International Conference on Control, Decision and Information Technologies (CoDIT (IEEE, 2014), 74–80.

[27] A. Azadeh , M. Hosseinabadi Farahani , S. Torabzadeh , and M. Baghersad , “Scheduling Prioritized Patients in Emergency Department Laboratories,” Computer Methods and Programs in Biomedicine 117, no. 2 (2014): 61–70. 25214024 10.1016/j.cmpb.2014.08.006

[28] T. Ren , B. Liu , P. Zhao , et al., “Two‐Stage Flow‐Open Shop Scheduling Problem to Minimize Makespan.” Intelligent Computing Theories and Application: 12th International Conference, ICIC 2016, Lanzhou, China, August 2‐5, 2016. Proceedings, Part I (Springer International Publishing, 2016), 527–535.

[29] A. Azadeh , M. Baghersad , M. H. Farahani , and M. Zarrin , “Semi‐Online Patient Scheduling in Pathology Laboratories,” Artificial Intelligence in Medicine 64, no. 3 (2015): 217–226. 26012952 10.1016/j.artmed.2015.05.001

[30] W. Tan , X. Yuan , G. Huang , and Z. Liu , “Low‐Carbon Joint Scheduling in Flexible Open‐Shop Environment With Constrained Automatic Guided Vehicle by Multi‐Objective Particle Swarm Optimization,” Applied Soft Computing 111 (2021): 107695.

[31] T. Gonzalez and S. Sahni , “Open Shop Scheduling to Minimize Finish Time,” Journal of the ACM 23, no. 4 (1976): 665–679.

[32] P. Papadopoulos , D. W. Coit , and A. A. Ezzat , “Seizing Opportunity: Maintenance Optimization in Offshore Wind Farms Considering Accessibility, Production, and Crew Dispatch,” IEEE Transactions on Sustainable Energy 13, no. 1 (2022): 111–121.

[33] M. E. Matta , “A Genetic Algorithm for the Proportionate Multiprocessor Open Shop,” Computers & Operations Research 36, no. 9 (2009): 2601–2618.

[34] D. Bai , Z. H. Zhang , and Q. Zhang , “Flexible Open Shop Scheduling Problem to Minimize Makespan,” Computers & Operations Research 67 (2016): 207–215.

[35] A. A. Rahmani Hosseinabadi , J. Vahidi , B. Saemi , et al., “Extended Genetic Algorithm for Solving Open‐Shop Scheduling Problem,” Soft Computing 23 (2019): 5099–5116.

[36] Z. Adak , M. Ö. Arıoğlu , and S. Bulkan , “An Ant Colony Optimization Approach for the Proportionate Multiprocessor Open Shop,” Journal of Combinatorial Optimization 43, no. 4 (2022): 785–817.

[37] L. R. Abreu , J. O. Cunha , B. A. Prata , et al., “A Genetic Algorithm for Scheduling Open Shops With Sequence‐Dependent Setup Times,” Computers & Operations Research 113 (2020): 104793.

[38] T. F. Abdelmaguid , “Bi‐Objective Dynamic Multiprocessor Open Shop Scheduling: An Exact Algorithm,” Algorithms 13, no. 3 (2020): 74.

[39] J. Behnamian , S. Memar Dezfooli , and H. Asgari , “A Scatter Search Algorithm With a Novel Solution Representation for Flexible Open Shop Scheduling: A Multi‐Objective Optimization,” Journal of Supercomputing 77 (2021): 13115–13138.

[40] M. Enayati , M. Yousefi Nejad Attari , and F. Lotfian Delouyi , “Optimizing Open Shop Scheduling: Minimizing Makespan Through Whale Optimization Algorithm and Transportation Time Consideration,” Journal of Quality Engineering and Production Optimization 8, no. 1 (2023): 133–150.

[41] J. Guo , B. Peng , B. Du , et al., “Q‐Learning‐Based Multi‐Objective Spotted Hyena Algorithm for Flexible Open Shop Scheduling Problem With Consideration of Preventive Maintenance and Travel/Setup Times,” Journal of Manufacturing Systems 82 (2025): 224–238.

[42] A. S. Kiran and M. L. Smith , “Simulation Studies in Job Shop Scheduling—II: Performance of Priority Rules,” Computers & Industrial Engineering 8, no. 2 (1984): 95–105.

[43] V. Selladurai , P. Aravindan , S. G. Ponnambalam , et al., “Dynamic Simulation of Job Shop Scheduling for Optimal Performance,” International Journal of Operations & Production Management 15, no. 7 (1995): 106–120.

[44] M. H. Kim and Y. D. Kim , “Simulation‐Based Real‐Time Scheduling in a Flexible Manufacturing System,” Journal of Manufacturing Systems 13, no. 2 (1994): 85–93.

[45] R. Tavakkoli‐Moghaddam and M. Daneshmand‐Mehr , “A Computer Simulation Model for Job Shop Scheduling Problems Minimizing Makespan,” Computers & Industrial Engineering 48, no. 4 (2005): 811–823.

[46] H. L. Lu , G. Q. Huang , and H. D. Yang , “Integrating Order Review/Release and Dispatching Rules for Assembly Job Shop Scheduling Using a Simulation Approach,” International Journal of Production Research 49, no. 3 (2011): 647–669.

[47] Y. Yan and G. Wang , “A Job Shop Scheduling Approach Based on Simulation Optimization.” 2007 IEEE International Conference on Industrial Engineering and Engineering Management (IEEE, 2007), 1816–1822.

[48] M. Gholami and M. Zandieh , “Integrating Simulation and Genetic Algorithm to Schedule a Dynamic Flexible Job Shop,” Journal of Intelligent Manufacturing 20, no. 4 (2009): 481–498.

[49] M. Frantzén , A. H. C. Ng , and P. Moore , “A Simulation‐Based Scheduling System for Real‐Time Optimization and Decision Making Support,” Robotics and Computer‐Integrated Manufacturing 27, no. 4 (2011): 696–705.

[50] S. Lang , F. Behrendt , N. Lanzerath , et al., “Integration of Deep Reinforcement Learning and Discrete‐Event Simulation for Real‐Time Scheduling of a Flexible Job Shop Production.” 2020 Winter Simulation Conference (WSC) (IEEE, 2020), 3057–3068.

[51] D. E. Goldberg , Genetic Algorithms in Search, Optimization, and Machine Learning (Addison‐Wesley, 1989).

[52] G. Syswerda , “Scheduling Optimization Using Genetic Algorithms.” Handbook of Genetic Algorithms (New York: Van Nostrand Reinhold, 1991).

[53] P. Larranaga , C. M. H. Kuijpers , R. H. Murga , et al., “Genetic Algorithms for the Travelling Salesman Problem: A Review of Representations and Operators,” Artificial Intelligence Review 13 (1999): 129–170.

[54] J. Powers , J. M. McGree , D. Grieve , et al., “Managing Surgical Waiting Lists Through Dynamic Priority Scoring,” Health Care Management Science 26, no. 3 (2023): 533–557. 37378722 10.1007/s10729-023-09648-1 PMC10484819

[55] A. Ala , V. Simic , M. Deveci , et al., “Simulation‐Based Analysis of Appointment Scheduling System in Healthcare Services: A Critical Review,” Archives of Computational Methods in Engineering 30, no. 3 (2023): 1961–1978.

[56] D. L. Olson , “Project Scheduling.” Project Management Tools. AI for Risks. Singapore (Springer, 2024).

[57] X. Chen , R. Duan , Y. Shen , et al., “Design and Evaluation of an Intelligent Physical Examination System in Improving the Satisfaction of Patients With Chronic Disease,” Heliyon 10, no. 1 (2024): e23906. 38192845 10.1016/j.heliyon.2023.e23906 PMC10772725

[58] X. Wang , R. Zhang , Z. Gao , et al., “Patient‐Centered Outpatient Process Optimization System Based on Intelligent Guidance in a Large Tertiary Hospital in China: Implementation Report,” JMIR Medical Informatics 13, no. 1 (2025): e60219. 40882153 10.2196/60219 PMC12396732

[59] M. Assel , D. Sjoberg , A. Elders , et al., “Guidelines for Reporting of Statistics for Clinical Research in Urology,” Journal of Urology 201, no. 3 (2019): 595–604. 30633111 10.1097/JU.0000000000000001 PMC6600813

